# Ultrasound measurements of interactive turn-taking in question-answer sequences: Articulatory preparation is delayed but not tied to the response

**DOI:** 10.1371/journal.pone.0276470

**Published:** 2023-07-05

**Authors:** Sara Bögels, Stephen C. Levinson

**Affiliations:** 1 Department of Communication and Cognition, Tilburg University, Tilburg, The Netherlands; 2 Max Planck Institute for Psycholinguistics, Nijmegen, The Netherlands; 3 Donders Institute for Brain, Cognition, and Behaviour, Radboud University, Nijmegen, The Netherlands; Universite Sorbonne Nouvelle Paris 3, FRANCE

## Abstract

We know that speech planning in conversational turn-taking can happen in overlap with the previous turn and research suggests that it starts as early as possible, that is, as soon as the gist of the previous turn becomes clear. The present study aimed to investigate whether planning proceeds all the way up to the last stage of articulatory preparation (i.e., putting the articulators in place for the first phoneme of the response) and what the timing of this process is. Participants answered pre-recorded quiz questions (being under the illusion that they were asked live), while their tongue movements were measured using ultrasound. Planning could start early for some quiz questions (i.e., midway during the question), but late for others (i.e., only at the end of the question). The results showed no evidence for a difference between tongue movements in these two types of questions for at least two seconds after planning could start in early-planning questions, suggesting that speech planning in overlap with the current turn proceeds more slowly than in the clear. On the other hand, when time-locking to speech onset, tongue movements differed between the two conditions from up to two seconds before this point. This suggests that articulatory preparation can occur in advance and is not fully tied to the overt response itself.

## Introduction

Imagine you are participating in a television quiz in which you have to answer several knowledge questions, each with a five second deadline. Listening to the quizmaster, you hear “Which character, also called 007…”. Even though it is clear that the question is not over yet, hearing only this first part of the question allows you to start retrieving the answer to the question. Will you indeed start retrieving the answer, instead of waiting for the end of the question? Or perhaps you will go even further and you will continue planning the answer (‘James Bond’) as far as positioning your tongue in the correct position, such that after the question is over (“… appears in the famous movies?”) you have already prepared your articulators and can utter the correct answer as fast as possible? The present study investigates, using ultrasound measures of tongue movements, whether speakers, when planning responses in advance, go so far as to prepare their articulators in advance as well, and if so, what the timing of this process is.

### Short gaps in conversation

When people take turns talking, whether during casual conversation or in a quiz or interview-like situation as in the example above, they generally do so quite smoothly, with mostly short gaps between two turns of different speakers [1]. Indeed, observational studies focusing on corpora of conversational speech show that the most frequent gap between turns is only around 200 ms long (e.g., [2, 3]). At first sight, 200 ms appears remarkably short when compared to, for example, laboratory studies on word production (mostly in picture naming contexts), yielding speaking latencies of 600 ms or longer [4]. Such short gaps in conversation strongly suggest that speech planning of a turn already starts during listening to the previous turn [5, 6]. However, several caveats should be noted around this puzzle, and have recently been put forward in the literature. First, laboratory production latencies are difficult to compare to conversational turns for many reasons (see [7]). For example, participants in laboratory studies are mostly asked to produce a single content word that is not contingent on previous speech, with a clear starting point for the reaction time (e.g., the onset of a picture), which is very different in conversation. Second, a large part of the turn transitions in conversational corpora take place before backchannels, continuations after such backchannels, or turns starting with fillers or particles [8], which are likely to require much less planning time than content words. Third, in many corpora used in studies that estimate turn timings, the turns and gaps are extracted automatically (e.g., [2, 9]), such that it is not clear whether only gaps between actual *turns* are counted. Relatedly, not all turns appear to be contingent on the previous turn, but a substantial amount of turns within conversational corpora, at least when they are automatically annotated, appear to be continuations of a previously started turn [10]. However, even when keeping these considerations in mind, one should note that even studies using carefully annotated corpora and/or considering only answers to questions (thus excluding backchannels and non-contingent turns) (see [3, 11, 12]), show the same typically reported distribution consisting of predominantly very short responses (i.e., around 200 ms). The fact that very fast responses are possible and abundant even in such samples thus still suggests that language production planning of contingent responses (e.g., to questions) often overlaps with the previous turn.

### Response planning starts early

Indeed, over the last decade several studies have empirically shown that production planning of the current turn can start in overlap with the previous turn. For example, studies using a competing dual-task while participants were taking turns with another, found that this competing task slowed down just before the end of the previous turn [13, 14], suggesting at least some overlap between speech planning and listening. In other experimental studies [15–19], some creating an illusion of a live interaction, participants were asked pre-recorded questions which allowed an early or a late start of response planning. An example of such an early-planning condition is given in example (1) below [17]; repeated from the example given above, and also part of the stimuli of the present study), with example (2) showing a late-planning condition. The critical word ‘007’ that allows participants to start planning their response, appears in the middle or at the very end of the question, respectively.

1. Which character, also called 007, appears in the famous movies?
2. Which character from the famous movies, is also called 007?

In all these studies, first, response times relative to the ends of questions were faster for early-planning (e.g., example 1) than for late-planning questions (e.g., example 2), again showing that, in the former case, at least part of response planning happens in overlap with the question [17]. In addition, an eye-tracking study [15] asked participants to listen to a confederate naming some objects, after which they themselves should name the rest of the objects in the display. The results showed that participants looked at the objects they needed to name as soon as the information in the previous turn led them to do so, suggesting they started planning the names for these objects as soon as possible. In addition, EEG measurements during interactive question-answer tasks, using questions such as examples (1) and (2) [17, 18], found a neural signature of response planning starting within half a second after the start of the critical word in both conditions (e.g., *007* in examples 1 and 2). This large positive-going waveform in the ERPs was associated with response planning because it was much reduced in a replication of the experiment in which participants did not respond to the questions, but instead were asked to remember them. Moreover, the positivity was replicated in a more natural, unconstrained interview-like setting as well [11]. A different experimental turn-taking study [20] asked participants to either answer simple yes/no questions or estimate the end of these questions using a button-press. The predictability of the question was manipulated and predictable questions (such as: “Are dogs your favorite animal?”) led to faster verbal responses (but not more precisely timed button-presses) than less predictable questions, thus suggesting that response planning starts already during the question in cases where the end of the question can be predicted.

### The costs of overlap

Together, the studies cited above show that there is often at least some overlap between planning of the upcoming turn and listening to the current one, while several of the studies strongly suggest that upcoming responders start planning responses as soon as they can. This could lead to substantial overlap between comprehension and production planning. However, such overlap may be costly since both of these processes require cognitive effort and may interfere with one another ([21, 22]; see [23] for a more elaborate discussion). Linguistic comprehension tasks have indeed been shown to interfere with language production planning, more so than non-linguistic tasks. For example, picture naming showed more interference from a simultaneous linguistic syllable judgment task, than from a non-linguistic tone judgment task ([24]; see also [25]). In a turn-taking setting [26], pupillometry measurements showed that response planning in overlap with the incoming turn resulted in a higher cognitive load than response planning in silence. This finding is consistent with the response time results from the studies described above [15–19], in that the gain in response time for early-planning questions was not as high as one may expect give the increase in planning time. For example, in [17], responses occurred on average about 300 ms earlier in the early-planning than the late-planning condition whereas the extra time to plan was on average 1700 ms. Even considering that participants may have avoided interrupting the question, the average response time for the early-planning condition was about 640 ms, so participants could have won more time if speech planning was as efficient as, for example, in picture naming studies (i.e., taking around 600 ms). This suggests that production planning proceeds slower and/or less efficiently when it is done in overlap with listening to the incoming turn. At the same time, comprehension of the incoming turn may also suffer when planning of the next turn is already ongoing. In an EEG study [18], it was shown that the N400 effect (i.e., the ERP difference between predictable and unpredictable words in a question) was smaller after planning had started (i.e., in early-planning questions, such as in example 1) than when it had not yet started (in late-planning questions, such as in example 2). However, this was only the case for participants with generally fast response times, who thus may have prioritized response planning over comprehension. Another study [27] investigated a similar question using semantic illusions, which are semantic inconsistencies that are often overlooked by listeners or readers, such as “How many animals of each kind did *Moses* take on the Ark?” (where *Moses* should be *Noah*). This study showed that listeners detected such semantic illusions less often when they were already planning their response than when they could not yet do so, also suggesting that they paid less attention to the questions when they were already planning. In sum, overlap between comprehension of the incoming turn and planning of one’s current turn is likely to be cognitively demanding and it has been shown that such overlap can lead to one or both tasks being done less efficiently and/or suboptimally.

### Stages of planning in overlap

From the literature reviewed so far, planning appears to start as early as possible, but proceed more slowly and/or less efficiently while still in overlap with the previous turn. However, what has been studied much less, is which stages of speech planning can be performed in overlap with the incoming turn; or how far the planning actually proceeds. This issue may also be relevant for the question of how costly the overlap is, since different stages may differ in the extent to which they are cognitively demanding and require attention (e.g., [28]). Language production models distinguish between different stages within speech planning [4, 29], such as conceptualization, lemma selection, word form retrieval, and phonetic and articulatory encoding. One of the EEG studies mentioned above [17] attempted to localize the positivity in the ERPs that they interpreted as the start of production planning. The effect was localized to brain areas that have been related to language production, including lemma retrieval and selection, phonological code retrieval, and syllabification. On the basis of these results, it was speculated that response planning during the incoming question may proceed up until later stages of, for example, phonological retrieval. However, one has to keep in mind that localization of effects in EEG is difficult due to distortion of signals by the skull (e.g., [30]). Moreover, the found areas have also been shown to be involved in other processes, such as language comprehension (e.g., [31]). A more recent EEG study [32], attempted to replicate these neural signatures using a simpler paradigm. Participants were asked to plan articulation of a nonword presented on the screen, but then withhold articulation until a go-signal appeared. In the waiting interval, a positivity was found in the EEG, reminiscent of the neural signature found in turn-taking EEG studies [17, 18]. However, crucially, the authors found a very similar neural response in another condition in which participants performed delayed lexical decision on the word (responding via a button-press) instead of pronouncing it. The authors argued that these neural responses were therefore unlikely to reflect (phonological) response planning, but instead might reflect monitoring for the go-signal, which happens in both conditions. However, it is highly unlikely that this is also the explanation for the neural signatures in the turn-taking EEG studies (e.g., [17]). In this study, a larger positivity was found for late-planning questions at question end (where planning starts for these questions), whereas monitoring for the turn end should take place at least to the same extent for early-planning conditions at this point. Still, the fact that no neural signature of phonological planning (which should be required for reading nonwords out loud) was found in this study with a simpler paradigm [32] suggests that it is hard to find such a signature and that the effects found in turn-taking contexts [11, 17, 18] may reflect earlier stages of response planning (that were not part of the task in [32]).

One recent study [23] investigated specific stages of response planning in turn-taking, using a task-switching approach. In a paradigm in which participants answered early-planning or late-planning questions by naming one of four pictures, they had to switch to another task in a quarter of the trials. In those switching trials, once participants had fixated the correct picture (e.g., the Dutch word *Appel*, ‘Apple’) for 300 ms (i.e., before uttering their response), the pictures disappeared and participants instead needed to perform lexical decision on a presented written word that was either phonologically related to the target picture that they were about to name (e.g., *Ampel*) or not. They found that phonological relatedness with the target picture facilitated reaction times in the lexical decision task. Thus, they concluded that phonological planning of the picture name was ongoing at this point in time, leading to facilitated recognition of the written word. Interestingly, this point (i.e., when participants fixated the correct picture for 300 ms) occurred on average more than a second after critical word onset in early-planning questions, which is quite a bit later than one would expect based on estimates from picture naming studies in isolation. Thus, this is once again an indication that speech planning proceeds more slowly while listening to the previous turn. However, this time point on average occurred still more than a second before question offset, suggesting that phonological planning of the response can occur in overlap with the incoming turn, and is not postponed until after its end.

### Articulatory preparation in overlap?

So far, research thus suggests that speech planning starts as soon as enough information is available, often in overlap with the ongoing turn, and then proceeds more slowly than in isolation but still possibly in overlap with the question at least until the stage of phonological planning. However, what about even later stages in the planning process? Do they also proceed during the incoming turn, or are some of these processes postponed until after the other’s turn is over? In the present study, we will focus on the very last stage before speech production, which we will refer to as ‘articulatory preparation’. By this term, we mean all motor actions of the articulators that prepare the speaker for actual speech (i.e., using sound to utter words). Thus, examples of such articulatory preparation may be breathing in before one speaks, silent mouth movements or mouth openings, and movements (e.g., of the tongue) that bring the articulators into position for the first phoneme. To our knowledge, only two earlier studies have considered the timing of such preparatory articulatory movements, one focusing on inbreaths and one on lip movements. The first study [33] investigated the timing of inbreaths before responses to questions within natural conversations between friends. When an inbreath occurred before a response, it was most often launched close to the offset of the question. This result would suggest that interlocutors postpone this type of articulatory preparation until they recognize imminent completion of the previous turn, as has been suggested for launching speech itself [12, 34]. This may imply that inbreaths are regarded as part of the turn itself and are generally drawn just before the turn, instead of longer in advance such that the upcoming speaker is prepared for articulation earlier. The second study that investigated the timing of articulatory preparation [35] looked at preparatory lip movements as captured with video recordings in a conversational corpus. They investigated how widely speakers opened their mouths in advance of a turn starting with a sound that requires a large versus a small mouth opening. This study found that differences in lip openings between these response types occurred up to three seconds before speech onset, especially for short turns. Given that gap sizes are usually much shorter than that, this result shows that articulatory preparation can occur well in overlap with the previous turn. However, one has to keep in mind that this study included all turns in the corpus, also backchannels and presumably turns that were a continuation of previously initiated turns, and not necessarily a response to the previous turn (see [10]). These two existing studies on the timing of articulatory preparation thus show conflicting results, that is, inbreaths appear to occur just before the start of the turn [33], whereas preparatory mouth movements can occur much longer in advance [35]. However, note that these are both observational studies that did not investigate in what way articulatory preparation was related to the moment that interlocutors could start planning their turn. The present study, by contrast, takes an experimental approach to this question, measuring articulatory preparation in the form of tongue movements, using ultrasound measurements, before responses to questions that allow early versus late planning (e.g., examples 1 and 2).

### The present study

The main aim of the present study is to investigate when during turn-taking upcoming speakers start with articulatory preparation of their turn; whether they do this ‘as early as possible’ and often still during the ongoing turn or whether such preparation is tied to the articulation of the response itself. In order to estimate articulatory preparation, we measure participants’ tongue movements using ultrasound. The paradigm and stimuli are taken from the earlier EEG study described above [17], that is, a quiz situation with early- and late-planning questions (e.g., examples 1 and 2 above). In that way, the timing of articulatory preparation can be compared between responses that can be planned midway through a question versus responses that can be planned only at the very end of the question.

**Fig 1:**
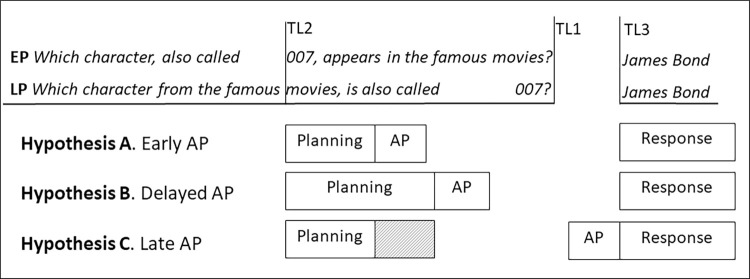
Illustration of hypotheses. Example of an early-planning question (EP; i.e., planning can start when listeners recognize the word *007* in the middle of the question) and a late-planning question (LP; i.e., planning can start when listeners recognize the word *007* at the end of the question) with indications of the three different time-locking points for the ultrasound analyses: question offset (TL1), early critical word onset (TL2), and speech onset (TL3). At the bottom of the figure, the three hypotheses for EP are schematically depicted (AP = articulatory preparation): Hypothesis A (Early AP) predicts that planning proceeds fast and immediately continues up to and including AP. Hypothesis B (Delayed AP) predicts that planning is less efficient and slower during turn-taking (than in isolation) but still immediately proceeds up until and including AP. Hypotheses C (Late AP) predicts that planning proceeds up to a certain point (i.e., it may be fast or slow, indicated by the shaded part of the box) but that (at least) AP is fully tied to the speech and occurs immediately before the response.

To analyze the ultrasound results, we compare average pixel hues between subsequent frames in the ultrasound video [36], creating a continuous trace reflecting tongue-movements over time. This trace can then be time-locked to different positions of interest and averaged over trials (as is common in ERP analyses of EEG data, for example). A first aim of the present study is to replicate earlier research that found faster response times (relative to question offset) for questions that allow for earlier planning. If the ultrasound method (and our chosen analysis) is valid, we should find a corresponding pattern of results when time-locking tongue-movements over time to question offset (see Fig 1, time-locking/TL1), that is, we hypothesize to see an earlier peak in the amount of tongue movement for the early-planning than the late-planning condition.

More importantly, we place a second time-locking point in the middle of the question (Fig 1, time-locking/TL2), at the onset of the critical word, enabling the start of production planning in the early-planning questions, and a word at an equivalent position in the middle of the question in late-planning questions. This was also the critical time-locking point for the EEG signal in [17], showing neural signatures indicative for response planning after only around 500 ms. If we assume that (the first stages of) response planning indeed start very shortly after the critical word is recognized, there are different options for how the next stages of planning proceed and ultimately when articulatory preparation is performed. First, let’s consider the extreme possibility that planning proceeds as fast as for picture naming in isolation (i.e., in total taking around 600 ms; [4]); this hypothesis is depicted in Fig 1 as hypothesis A (Early articulatory preparation/AP). In that case, articulatory preparation (AP) might start somewhere around 600–900 ms after critical word onset (assuming word recognition takes about 300 ms). This would predict differences between the two conditions within a second after critical word onset. However, given the results discussed above, suggesting that speech planning appears to proceed more slowly or less efficiently during an incoming turn [15–19], it is realistic to expect (much) later effects, even if planning immediately proceeds all the way up to articulatory preparation. If we can rely on the results of [23], although they used a different task, the stage of phonological planning might be going on around 1000 ms after critical word onset, which makes it most likely that articulatory preparation will start even later (i.e., not before one or even two seconds after critical word onset). This hypothesis is depicted in Fig 1 as hypothesis B (Delayed articulatory preparation/AP). Note that this hypothesis generally predicts that the planning process is ‘drawn out’ or takes longer than in isolation but makes no specific claims regarding which phases of the planning process take longer, or how much longer.

On the other extreme of the spectrum, articulatory preparation may be fully tied to production of the response itself. To examine that possibility, we also time-lock the ultrasound changes to response onset (time-locking/TL3 in Fig 1) and look backwards. If articulatory preparation is indeed locked to production, meaning that response production goes ballistic from the moment that articulatory preparation has started, we should not expect to see differences between early- and late-planning questions for this time-locking point (see Fig 1, hypothesis C: Late articulatory preparation/AP). In contrast, when tongue movements start longer before response planning in the early-planning than in the late-planning condition, this implies that articulatory preparation starts longer before articulation when there is more planning time, which means the two processes are not fully tied to each other.

## Materials and methods

### Ethical approval

The study was carried out in accordance with guidelines approved by the Ethics Committee Faculty Social Sciences of the Radboud University Nijmegen.

### Participants

Thirty participants, recruited from the participant database of the Max Planck Institute for Psycholinguistics, Nijmegen, took part in the experiment (8 male, 22 female) with an average age of 21.8 years old (*SD* = 1.86; range: 19–26). All participants were native speakers of Dutch and did not have any hearing disorders. Participants were paid 10 euros per hour (i.e., 10–12 euros in total depending on the precise length of the experiment). Five participants were removed before the analysis, two because of insufficient quality of the ultrasound recording, two because of an excessive number of hesitations, leaving too few trials for analysis, and one because of excessive movement of the ultrasound probe. The final set of 25 participants (6 male, 19 female) had a mean age of 21.8 years old (*SD* = 1.85, range: 19–26).

### Materials

The experimental stimuli used in the experiment were a subset of the quiz questions used in [17], each in both an early-planning and a late-planning condition (see examples 1 and 2 in the Introduction and in Fig 1). From the original 96 quiz questions used in that study, a subset of 58 questions was selected (some were slightly adapted) of which the answer started with a phoneme that can be picked up well by the ultrasound method [37], that is, excluding the Dutch sounds d, f, ɣ, ɦ, m, p, r, v, and ʋ. Another reason for reducing the number of items in the experiment was that the ultrasound stabilization headset (see below) was rather heavy for participants, rendering an experiment length of more than 30 minutes undesirable. The early planning questions all contained a critical word (the onset of which constituted TL2, see Fig 1) that enabled participants to start planning their answer to the question, as verified in a pilot reported in [17]. For the late planning questions, a word at an equivalent position in the question was chosen (as in [17]) to compare the critical words to in TL2 (see Fig 1). These ‘control words’ were never the first or the last words in the question. Note that both the critical and control ‘words’ sometimes consisted of two or three words, for example if it constituted a (first and last) name. Table 1 lists several characteristics of the critical and control words in terms of position (relative to question onset/offset), word class and prosodic features. Given the naturalistic nature of the stimuli, the critical and control words are not matched perfectly, but most characteristics are relatively similar overall. All experimental stimuli (in Dutch), including information about the critical and control words can be found in Table A in S1 Text. In addition to these 58 experimental questions, 30 filler questions (i.e., more difficult questions) and 12 practice questions were also taken from [17], without any restrictions on the answer sounds since these were not analyzed.

**Table 1:** Characteristics of the critical words (within questions of the early-planning condition) and control words (within questions of the late-planning condition).

	Critical word (early-planning)	Control word (late-planning)
Question onset–word onset	*M* = 1619 ms, SD = 740	*M* = 1788 ms, *SD* = 950
	range: 137–3371 ms	range: 190–1245 ms
Word onset–question offset	*M* = 2839 ms, *SD* = 655	*M* = 2669 ms, *SD* = 866
	range: 1894–5501 ms	range: 1245–5274 ms
Pitch accented word	N = 58 (100%)	N = 57 (98%)
Last word before prosodic boundary	N = 47 (81%)	N = 42 (72%)
Last pitch-accented word before prosodic boundary	N = 55 (95%)	N = 48 (83%)
Word class		
Noun	N = 36 (62%)	N = 37 (64%)
Name	N = 11 (19%)	N = 6 (10%)
Verb	N = 7 (12%)	N = 6 (10%)
Adjective	N = 2 (3%)	N = 7 (12%)
Other	N = 2 (3%)	N = 2 (3%)

All items were recorded in the room from which the experiment leader addressed the participant (adjacent to the room of the participant, see *Procedure* below), spoken by the experiment leader, who also acted as ‘quiz master’ in the experiment. Pilot recordings ensured that the recorded questions sounded very similar to live speech sent from this same room through the sound system to the participant room.

### Design

Two stimulus lists were created with half of the (experimental and filler) items in the early-planning condition and the other half in the late-planning condition, switching the conditions between the two lists. Each list was administered to half of the original participants (11 and 14 of the 25 participants entering the analyses). The order of items was randomized and the same for all participants (as in [17]).

### Apparatus

**Fig 2:**
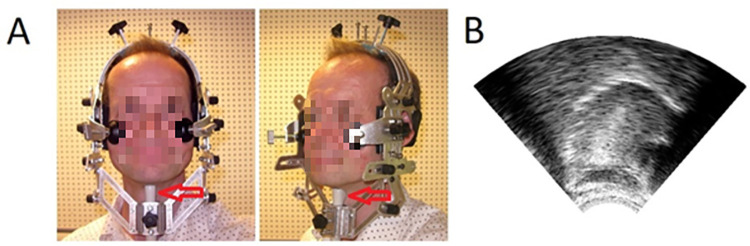
Ultrasound apparatus and example image. Panel A shows the stabilization headset for keeping the probe in place under the chin (see red arrow). Panel B shows an example of the fan-shaped ultrasound image with the tongue in mid-sagittal plane, as rendered by the ultrasound probe.

A Micrus Ext-1H ultrasound probe (Telemed UAB, Fig 2, red arrow in panel A) was used for the ultrasound measurements. It had a frame rate of 91.65 frames per second and produced a fan-shaped image of 57 by 842 pixels (see Fig 2, panel B). The probe was held in place under the chin by a stabilization headset (Articulate Instruments; Fig 2, panel A). In addition, A PS Stretch device was used to synchronize the ultrasound recordings with the presentation of the stimuli, sending a pulse to the ultrasound videos at the start of each auditory stimulus (i.e., recorded question).

### Procedure

In the recruitment email for the study, potential participants were informed that the experiment would involve ultrasound measurements, measuring an aspect of their speech, and the wearing of a kind of helmet, which would feel heavy after some time and possibly cause some discomfort. They were asked to take this information into consideration before signing up.

On the experiment day, participants were brought to the experiment room, where they first read and signed the informed consent form. Following, the experimenter put on and adjusted the stabilization headset to the participant and inserted the ultrasound probe. Gel was used between the probe and the participant’s skin to improve conduction. The probe was adjusted individually per participant to capture the tongue shape as well as possible. For this purpose, the ultrasound image was displayed on the screen in front of the participant in the experiment room; this screen was switched off afterwards. Then, the participant was asked to say *ta* and *ka* for baseline ultrasound measurements.

Participants were instructed to sit up straight during the experiment and try to keep looking at the same position on a black screen. The exact position was adjusted for each individual participant, based on their height. Furthermore, they were asked to make themselves as comfortable as possible and act as they would normally do. Regarding the quiz, participants received the same instructions and cover story as in the earlier EEG study [17]. That is, they were told that the quizmaster was asking the questions live in the next room, while in reality the prerecorded questions were played by the experimenter over speakers. They were informed that some questions would be very easy, but others would be harder and that they needed to answer within five seconds or their answer would count as incorrect. They would then receive immediate feedback from the experimenter (as in [17]). They were told that the two participants who answered most questions correctly would receive a prize. The experiment started with a practice block of 12 questions after which the participants were given the opportunity to ask questions. Then, the 88 experimental and filler questions were presented, divided into three blocks. Participants were given the opportunity to take a break in between blocks for as long as they wanted. They could drink some water with a straw. The experiment itself (excluding set-up and taking off the headset) took about 30 minutes.

During the experiment, the experiment leader sat in the room next to the experiment room and viewed the ultrasound image on a monitor. Articulate Assistant Advanced software (AAA, version 217.01; Articulate Instruments) was used to play the recorded wav-files, record the ultrasound measurements, and record each entire trial (i.e., the question, the answer, and the feedback) in a wav-file, to allow for response-time measurements. The experimenter controlled the timing of the question presentations. At the onset of each recorded question, a pulse was sent to the ultrasound measurements. After the participant answered (or when they did not respond for five seconds), the experimenter gave immediate live feedback to the participant via the sound system, indicating if the question was answered correctly and if not, what the right answer should have been.

After the end of the experiment, the experiment leader removed the ultrasound probe and the stabilization headset. Then, participants were asked to fill out a short questionnaire on paper. One side of the sheet of paper contained three questions regarding what they thought the purpose of the experiment was, whether they noticed anything during the experiment, and their experiences regarding the ‘helmet’ (stabilization headset). On the other side of the sheet of paper, they were asked two more explicit questions about the cover story: whether they thought that parts of the experiment may not have been live (and which parts) and whether, if yes, they already thought that during the experiment. Finally, they read a debriefing text, informing them about the fact that the quiz questions were prerecorded, why this was necessary, and explaining the purpose of the experiment. Subsequently they were given the opportunity to ask questions to the experiment leader.

### Data analysis

Before analyzing the behavioral (response time) and ultrasound data, trials with incorrect responses were removed, including those with a response time of longer than five seconds (*n* = 117; 8.1%) and correct responses including hesitations (*n* = 138; 11.5% of correct answers). Answers were counted as containing a hesitation when the correct answer was preceded by something else (e.g., ‘uh’ or a wrong answer), but not when the correct answer was spoken with a questioning or doubting intonation. Furthermore, responses starting with a sound hard to pick up with ultrasound (i.e., d, f, ɣ, ɦ, m, p, r, v, and ʋ; see materials above), for example because the answer was preceded by an article, were also removed (*n* = 58, 4.9% of correct answers without hesitations). This resulted in an average of 23.4 trials (*SD* = 2.29, range: 20–29) remaining per participant for the early-planning condition and 22.1 trials (*SD* = 2.16, range: 18–25) for the late-planning condition that were included in the analyses.

Given known inter-individual variability in the quality of ultrasound recordings (e.g., [36]), two coders (i.e., the first author and the experiment leader) scored the quality of one image of each participant on a scale of 0 (bad) to 2 (good) on two dimensions: the contrast of the tongue shape and the coverage of the tongue within the image. Averaging the scores of the two coders, seven participants scored below 1 on the contrast dimension, and six participants scored below 1 on the coverage dimension. Separate analyses were performed on the data excluding these seven and six participants in turn, in order to make sure that the results were not caused by artifacts present in any of these participants with potentially lower data quality (reported in Section B of S1 Text).

Behavioral response-time data were obtained using the speech-annotation software Praat [38], measuring the time between the onset of the participant’s response and the offset of the question in the wav-files created during the experiment. These data were analyzed using the lme4 package in R [39] using a linear mixed-effects model, with response time as the dependent variable, condition (early-planning, late-planning) as the main predictor and random intercepts for participant and item (since adding random slopes to the model led to a singular fit). For model coefficients, |*t*| > 2 was interpreted to correspond to significance at the 5% level (via the convergence of the t-distribution to the normal for large samples [40]).

The raw ultrasound data, that is, grayscale pixel hues of 57 by 842 pixels per frame (with values between 0: black and 255: white), were loaded into Matlab and the Euclidian distance was calculated between the pixel hues of subsequent frames per trial (also called the ’Delta technique’; [36, 41, 42]). This enables one to create a time series of relative change in the ultrasound image. Given that this data resembles EEG data in terms of its time series, the Matlab-based Fieldtrip software package for analyzing EEG data [43] was used in the next steps of the analysis. First, the time series were time-locked to three different positions in the trial: (1) question offset (i.e., TL1 in Fig 1), (2) the position in the question that planning could start (onset of critical word) in the early-planning condition and a word at an equivalent position in the middle of the question in the late-planning condition (i.e., TL2 in Fig 1, see also [17]), and (3) response onset (i.e., TL3 in Fig 1). Trials of the same condition were averaged per participant. Differences between conditions were then analyzed within a time window from the start until the end of the trial. Note that the length of the trials varied, such that the noise becomes larger at the beginnings and ends of the time windows, because fewer trials contribute to those timepoints. For statistical analysis, we used cluster-based analysis [44] implemented in Fieldtrip (as in [17]). This robust method reduces the multiple-comparisons problem and controls family-wise error across participants in time, given the many time-points in the time series. Paired t-tests are performed between the two experimental conditions for each time-point with a p-value threshold of .05. All time-points below the threshold are selected and clustered in time. Cluster statistics are calculated for each cluster by taking the sum of t-values in that cluster. To obtain a p-value for each cluster, a Monte Carlo method is used to estimate the permutation distribution of the largest cluster statistic. The permutation distribution is created by 1000 random permutations of the samples of the two conditions. At each randomization, clusters are identified and the largest sum of t-values of the clusters enters the permutation distribution. The proportion of maximum cluster statistics of the permutation distribution that is larger than the observed one is the p-value. The threshold was fixed to *p*  =  .017 (.05/3) to correct for the three analyses performed on the data within the three time-locking points.

## Results

### Questionnaire

In the post-experiment questionnaire, when asked if they noticed anything during the experiment, none of the participants mentioned potential pre-recording of questions. When asked explicitly whether some parts of the experiment may have been prerecorded, eight out of the 25 participants who entered the analysis indicated that the questions may have been prerecorded. Four of those indicated they already thought about that during the experiment. Separate analyses were performed on the data excluding the eight participants flagged in the first question, in order to exclude the possibility that the results would be unduly affected by participants that might have suspected that the questions were prerecorded (these control analyses are reported in Section B of S1 Text).

### Behavioral results

**Fig 3:**
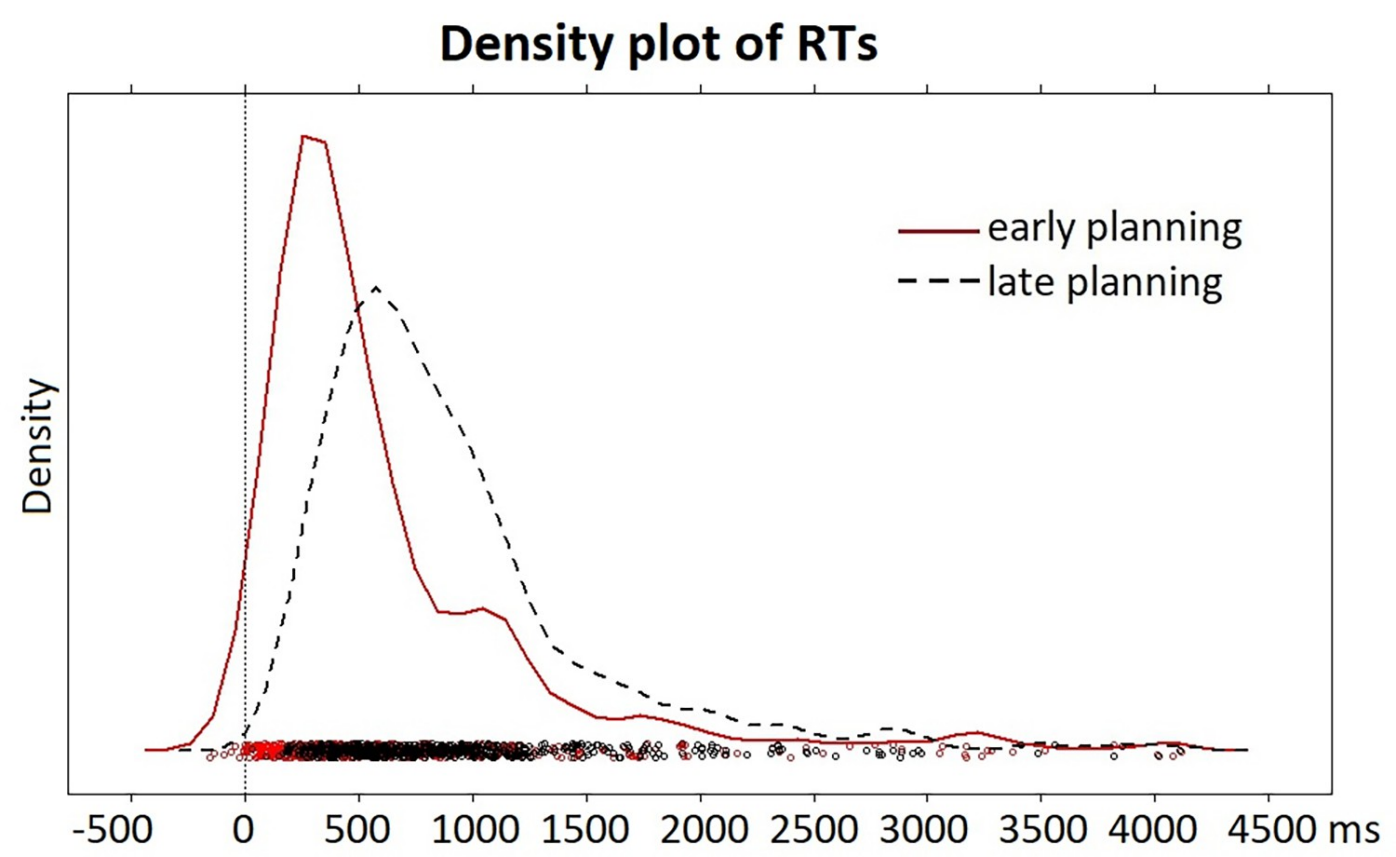
Behavioral results. Density plot of response times relative to question offset (TL1 in Fig 1) for questions where planning could start early (red solid line) and questions where planning could start late (black dashed line).

Fig 3 shows the distributions of response times to the offset of the questions, for the early-planning and late-planning conditions separately. As expected, the distribution for the early-planning condition is clearly shifted to the left (i.e., towards earlier responses) relative to the distribution for the late-planning condition, with modes slightly over 250 and 500 ms, respectively.

A linear mixed-effects model, with response time as the dependent variable, condition (early-planning, late-planning) as the main predictor and random intercepts for participant and item, showed that responses to questions in the early-planning condition (*M* = 626 ms; *Median* = 407 ms; *SD* = 662 ms) were indeed faster than responses in the late-planning condition (*M* = 900 ms, *Median* = 741 ms; *SD* = 617 ms; early-planning: *β*  =  669; late-planning: *β*  =  953; *t*  =  9.09).

### Ultrasound results

#### Question offset

**Fig 4:**
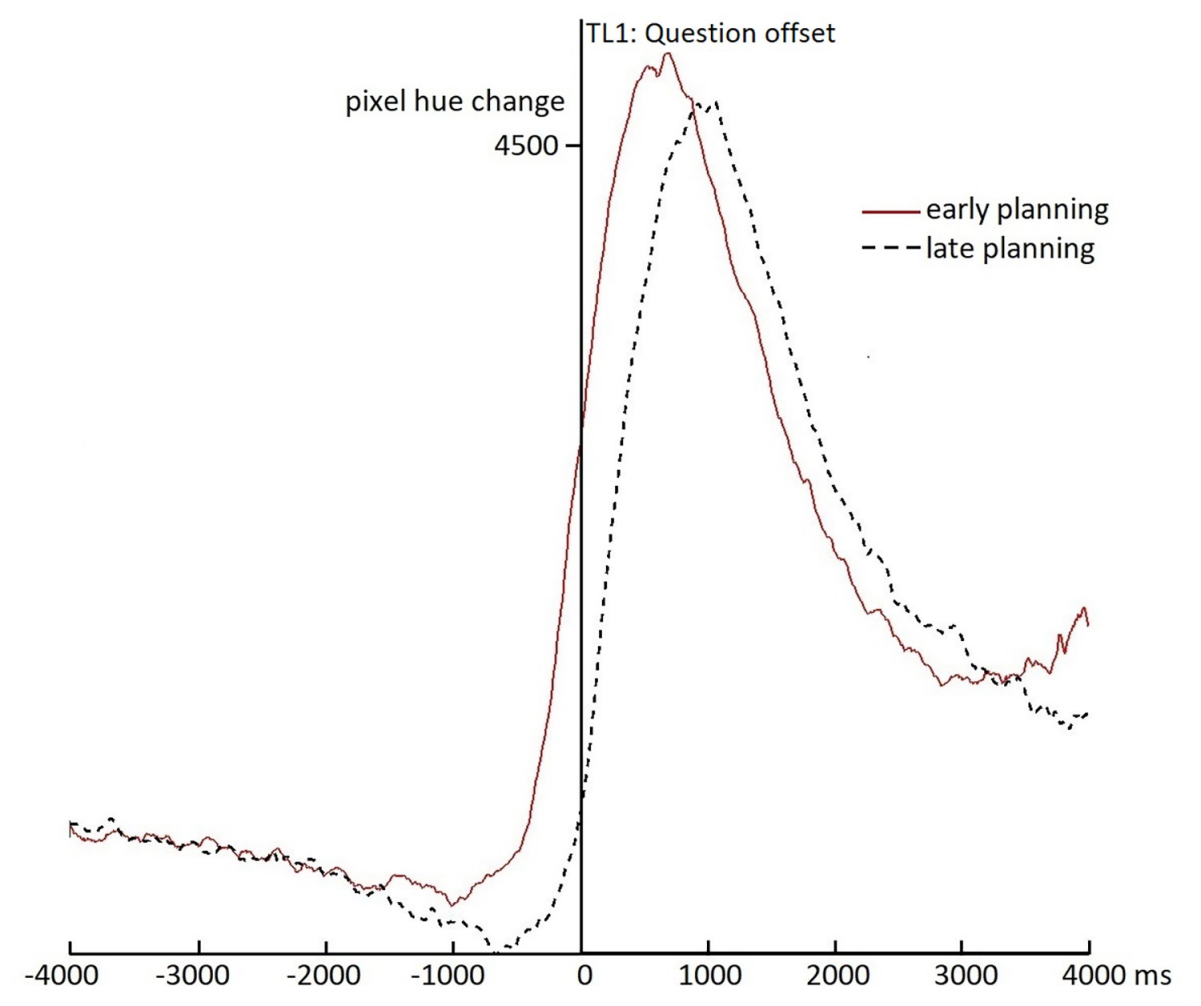
Ultrasound results for question offset. The figure shows frame-to-frame changes in pixel hues for subsequent ultrasound images relative to question offset (0-point, TL1 in Fig 1) for the early-planning (red solid line) and late-planning (black dashed line) conditions.

Fig 4 shows the frame-to-frame changes in the ultrasound image, reflecting the relative amount of movement of the tongue over time, time-locked to question offset (i.e., TL1 in Fig 1). This time-locking point is the same as in the behavioral response time results (cf. Fig 3) and was chosen as a sanity check to see if the ultrasound results would reflect the behavioral results. One may expect the largest movement of the tongue when participants are actually giving their response. Indeed, Fig 4 shows a peak in the amount of movement around the same time of the peak in response times in Fig 3 (and thus also earlier for the early-planning than the late-planning condition). Furthermore, Fig 4 shows an increase in tongue movement starting only shortly before question offset, also starting earlier in the early-planning than in the late-planning condition. This is confirmed by the cluster-based analyses, which show a larger amount of change for the early-planning than the late-planning condition between 884 ms before until 764 ms after question offset (*SumT* = 1133.0, *p* < .001). This effect is consistent with the behavioral results of earlier responses in the early-planning versus the late-planning condition, relative to question offset. In addition, there is an opposite effect between 993 and 2313 ms after question offset (*SumT* = -454.3, *p* = .003), visible in Fig 4 as the early-planning condition returning back to baseline earlier, which is probably due to the fact that responses generally also end earlier in this condition. A last observation from Fig 4 is that tongue movement appears to diminish while listening to the question until the moment that speech is initiated; that is, both lines descriptively slope downwards until just before the sharp rise towards the speech peak (i.e., around -1000 ms for the early planning condition and slightly later for the late planning condition). This may be due to participants avoiding any tongue movements (e.g., occasional swallowing) when they anticipate that they have to speak soon (we will come back to this observation in the Discussion).

#### Early planning point

**Fig 5:**
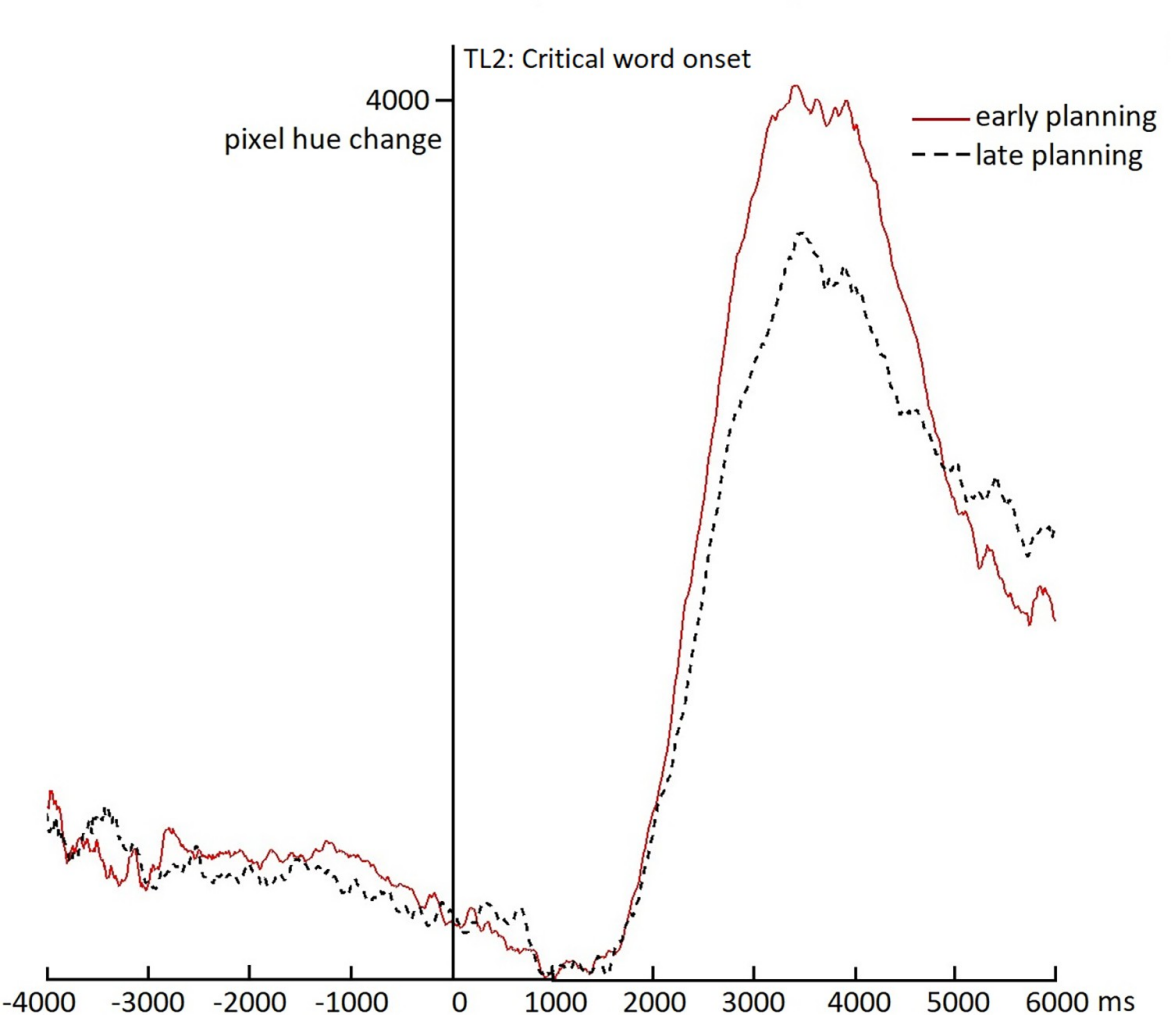
Ultrasound results for critical word onset. The figure shows frame-to-frame changes in pixel hues for subsequent ultrasound images relative to a position in the middle of the question (0-point, TL2 in Fig 1). For the early-planning condition (red solid line), the 0-point corresponds to the onset of the critical word that enables the start of response planning. For the late-planning condition (black dashed line) the 0-point corresponds to the onset of a word at an equivalent position in the middle of the question, where planning cannot start yet.

Fig 5 shows the tongue movement change over time for the early-planning and late-planning conditions, time-locked to the onset of the critical word in the early-planning condition and an equivalent position in the middle of the question in the late-planning condition (i.e., TL2 in Fig 1). That is, in the early-planning condition, participants can start planning their response after recognizing the word that started at time 0, whereas in the late-planning condition, participants cannot start planning at that same moment, but can start planning only much later, at the end of the question. Fig 5 shows no clear differences between the two conditions until at least two seconds after the time-locking point. Indeed, cluster-based analyses show a difference in tongue movement between 2193 and 4670 ms after the early planning point (*SumT* = 1036.1; *p* < .001). However, Fig 5 also suggests that tongue movements in both conditions start increasing at a very similar moment after the time-locking point (i.e., even before 2 seconds), but that the early-planning condition mainly reaches a higher peak, which causes the difference between the conditions. This higher peak may be due to a smaller variability in response times in the early-planning condition. This interpretation is corroborated by the response time results visible in Fig 3 which also show a higher and narrower peak in the early-planning than in the late-planning condition. In any case, no evidence is found that participants start preparing their articulators (i.e., their tongue) shortly (e.g., within 1000 ms, or even within 2000 ms) after they can start planning their response. While this null-result is no direct evidence for the absence of an early difference between the conditions, the data in Fig 5 do not show any indication for even a trend towards more tongue movements in the early than the late planning condition before 2000 ms (in fact between 0 and 1000 ms, descriptively more movement is visible in the late planning condition). Thus, we find no support for the Early AP hypothesis (hypothesis A in Fig 1). Instead, these results suggest that either articulatory preparation is strictly tied to the start of response planning (hypothesis C in Fig 1) or the response planning process is very much drawn out (hypothesis B in Fig 1).

#### Speech onset

**Fig 6:**
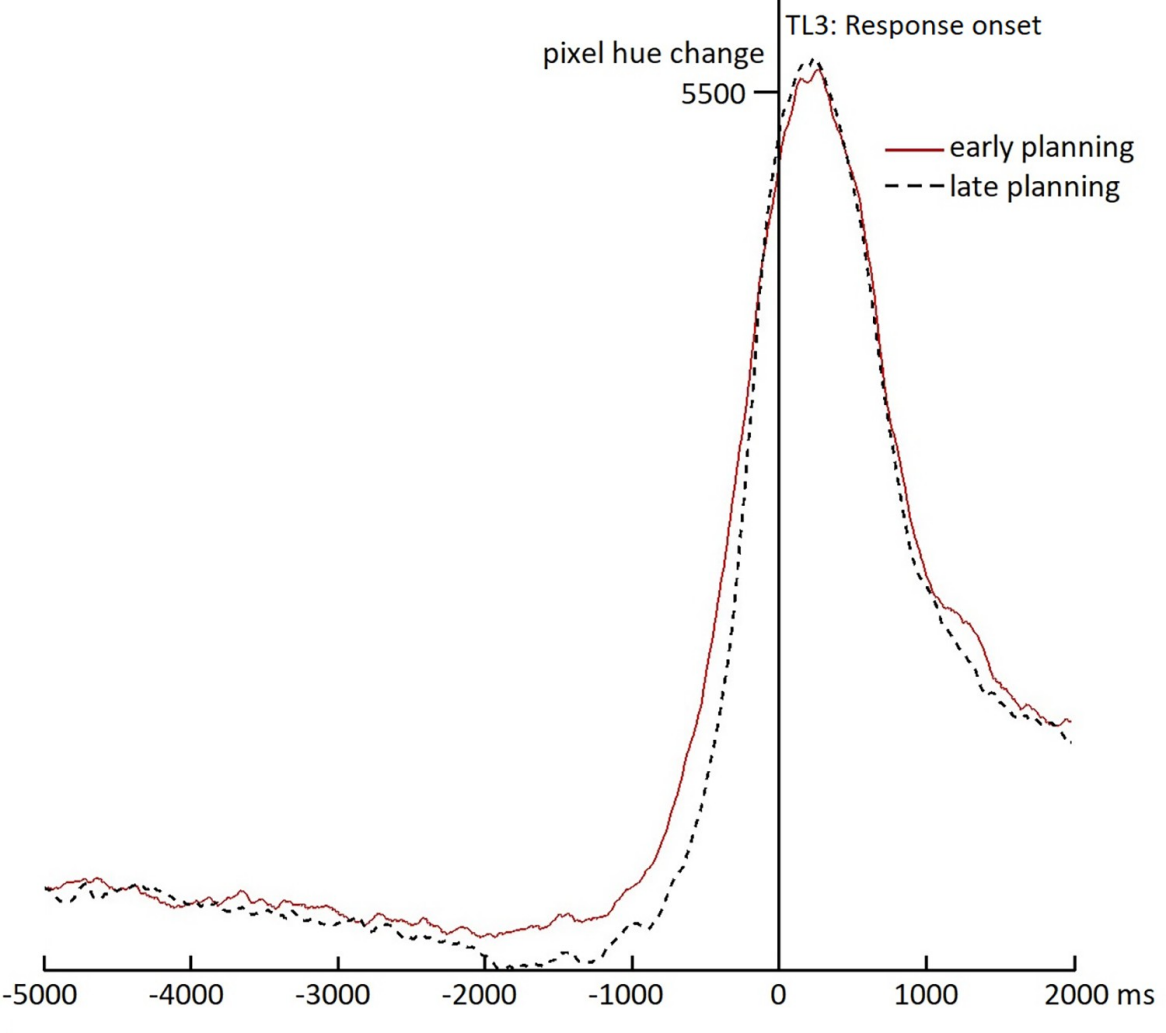
Ultrasound results for response onset. The figure shows frame-to-frame changes in pixel hues for subsequent ultrasound images relative to response onset (0-point) for the early-planning (red solid line) and late-planning (black dashed line) conditions.

Fig 6 shows the tongue movement change over time for the early-planning and late-planning conditions, relative to response onset as the zero-point (TL3 in Fig 1). Both conditions peak just after speech onset, when the largest tongue movements can be expected. More interestingly, around the base of the peak, a small difference is visible between the two conditions, suggesting that participants start preparing their tongue to speak earlier, relative to speech onset, in the early-planning than in the late-planning condition. Indeed, cluster-based analyses show a difference between the early-planning and the late-planning condition between 1920 and 153 ms *before* speech onset (*sumT* = 690.7; *p* < .001). This result shows that articulatory preparation (of the tongue) does not appear to be tied perfectly to articulation (as Hypothesis C in Fig 1 would predict), but that it can start up to two seconds beforehand, if the response can be planned in advance (as in the early-planning condition). Together with the results in the previous section, this thus supports the delayed articulatory preparation hypothesis (Hypothesis B in Fig 1).

## Discussion

The goal of the present study was to investigate the time course of articulatory preparation, as measured using tongue movements made visible with ultrasound, the latest stage of language production planning, during turn-taking. Assuming, based on earlier research (e.g., [17]), that response planning in turn-taking starts as early as possible, we were interested to see whether this response planning proceeds up to and including articulatory preparation in a turn-taking context, or whether planning only proceeds up to a certain point and articulatory preparation is rather tied to the vocal response itself.

First, the behavioral results show very similar results to earlier studies, most notably the EEG study from which the stimuli were taken [17]. Similar to that study, the present study showed faster response times for early-planning (*M* = 626 ms in the present study; *M* = 640 ms in [17]), than late-planning questions (*M* = 900 ms in the present study; *M* = 950 ms in [17]). The averages are also very comparable, suggesting that the ultrasound measurements (and the stabilization headset) did not impede responding, at least not more than an EEG cap. Furthermore, the ultrasound results time-locked to question offset (see Fig 4) are compatible with the behavioral results, showing a clear peak in tongue movements (corresponding to the articulation of the response) that is earlier for the early-planning than the late-planning condition. This suggests that ultrasound measurements are a feasible method for measuring movements related to speech in turn-taking situations and that the chosen analysis method (looking at changes in tongue movements based on changing pixel hues in the ultrasound image [36, 41, 42]) is valid in such a turn-taking context. Still, one should keep in mind that being able to pick up large movements during articulation does not necessarily imply that smaller preparatory movements can be picked up equally well in noisy circumstances. A second interesting observation that might be drawn from Figs 4–6 (but has not been quantified in the present study) is that the amount of tongue movements in both conditions generally appears to decrease during the course of the question until just before response onset. This observation is reminiscent of another observation within unpublished data from a dataset measuring breathing within conversation [33], which suggests that inbreaths become less frequent in the course of listening to a turn (i.e., people appear to ‘hold their breath’ while listening to the turn). It appears as if the articulators and speech system are generally being prepared for articulation by keeping them as still as possible, while it is not yet possible to know what the response should be. After all, it may take more time to articulate a response if one was just in the middle of swallowing or taking an inbreath. This observation may also relate to earlier work that uses real-time magnetic resonance imaging of the vocal tract, on the difference between ‘speech-ready’ and rest positions of the articulators [45, 46], suggesting that speech-ready positions (as well as inter-speech pauses) may be more mechanically advantageous for articulation than rest positions because they display less variability and increase the degree of active control over the speech planning process. However, the speech-ready position in those studies was defined as the last 200 ms before the start of an utterance, while the observations in the present study appear to span a larger period before speech onset. Future analyses of the present or related datasets could look more systematically into such ‘speech-ready’ positions in a turn-taking context and how far in advance of speech they may occur.

More importantly for the timing of articulatory preparation, the results showed no indication that response planning proceeds rapidly as fast as it can all the way through articulatory preparation (as e.g., in immediate picture naming; [4]), given that we found no evidence that the amount of tongue movements differed between early- and late-planning conditions until at least two seconds after onset of the word enabling response retrieval. On the other hand, the results also do not support a tight connection between articulatory preparation of the tongue and the response moment itself, given that we see more tongue movements when planning can start earlier from about two seconds before response onset. In other words, neither an ‘as fast as possible’ strategy nor a ‘wait till delivery’ strategy seems to account for the data. Rather, it appears that response planning is very much drawn out when responders are still listening to the previous turn. Thus, early phases of planning (such as conceptualization and memory retrieval) may start immediately when enough information about the gist of the current turn is available, while the latest stage (i.e., articulatory preparation), may occur only several seconds later. A related, but slightly different explanation of these results would be that planning proceeds up to, but not including articulatory preparation and that this last stage is only triggered by, for example, being able to predict when the question ends. In our late-planning condition, such prediction is probably not possible before response planning has ended, but in our early-planning condition planning might proceed up to articulatory preparation before the question end can be predicted. Note that both explanations imply that articulatory preparation is not tied to the response, but they differ regarding the trigger of articulatory preparation: either having finished all other stages of planning or being able to predict the question end. Future research may shed more light on this subtle question, for example by carefully manipulating the (timing of the) predictability of question ends.

We want to note here that our analyses could not directly prove the absence of an early articulatory preparation effect, so they are not fully conclusive in this regard. However, the results could not be attributed to poor quality of the ultrasound images or to participants suspecting that the questions were pre-recorded, given that the results were qualitatively very similar when excluding either participants with lower quality images or participants who may have guessed the recording of the questions. Despite the fact that not even a trend towards an early articulatory preparation effect was visible (if anything, it would be an opposite trend, see Fig 5), we cannot exclude the possibility that the present study lacked the power to detect potentially subtle differences in articulatory preparation between the two conditions at this early stage. Given the novelty of using ultrasound in relatively free circumstances, it was not deemed feasible to perform a power analysis. Future analyses of the present or similar data may use potentially more sensitive measures for analyzing ultrasound data, such as optical flow analyses (looking at directions of change in tongue movements) [47], which would require taking into account which phoneme is being prepared in which trial. Moreover, it is likely that the relatively naturalistic quiz questions used as stimuli were somewhat variable in their difficulty, which could have affected the timing of the planning process. Although the questions were pretested to be easy (by [17]), such that the majority of participants would answer them correctly, they may still vary with respect to how quickly the concept is retrieved or the lemma of the word can be accessed by participants. Similarly, these processes may be subject to individual variation between participants. It can be expected that early speech planning processes (as presumably reflected by the EEG signatures found in [17]) still show relatively little variation, but the variability increases as the planning process unfolds. Such potentially high variability in the timing of articulatory preparation may have obscured effects in the data, because the time-locking becomes suboptimal. Unfortunately, it is not possible to quantify this variability in the present study. Future research may attempt to diminish this variability by making the task even easier, for example by showing two pictures, one of which constitutes the answer to the question (cf. [18]).

Relating these results to previous research, they fit well with the findings from the dual task study described in the Introduction [23], showing that phonological planning, an intermediate stage in language production planning, appears to be ongoing around one second after the moment that planning can start (which is also around a second before question offset in that study). Combining these results with the present results and those of [17] and others described in the Introduction, one could paint the following picture. The early stages of response planning in turn taking appear to start as soon as possible, sometimes several seconds before question offset. Then, planning slowly proceeds up to phonological planning, taking place roughly a second later, and then proceeds (still slowly) until the last stage, articulatory preparation, taking place more than a second after that, but still possibly more than a second before the response itself (and therefore often starting well before question offset). As discussed above, it is also possible that this last stage is triggered by something else, such as being able to predict the end of the turn. Of course the precise timing of all these stages is likely to depend on several different factors, to which we will return below.

Comparing the present results to those from [35], on articulatory preparation (in the form of lip openings) in spontaneous conversation, one can say that the overall conclusions of the two studies appear compatible with each other. In a video corpus of spontaneous conversation, these authors compared how far participants opened their lips in advance of turns starting with sounds that required large versus small lip openings. Although the used measure was quite different from that used in the present study, both opening the lips and moving the tongue are actions that are likely to be performed in order to prepare for articulation of certain speech sounds or phonemes. The study [35] found that articulatory preparation could start several seconds before a response, especially for short responses. These effects even were present even longer before the response than in the present study, as early as three seconds before (compared to up to two seconds in the present study). However, one should keep in mind that the turns investigated in that study were very different from the ones in the present study. The present study looked at answers to open quiz questions only, which presumably require quite some amount of planning time (including retrieval of the response from memory), whereas the study by Krause and Kawamoto [35] considered all turns in a spontaneous corpus. The latter would include a large variety of turns, such as backchannels and continuations after backchannels. For these turns, it is unknown how early speakers can start planning, but earlier work suggests that at least responses to informal questions in a conversational context can often be planned after only a small part of the question has been heard [11]. Moreover, it has been argued that spontaneous conversation may also include turns (or turn constructional units) that are continuations of an earlier turn by the same speaker and may thus be (partly) planned even before the previous turn [10]. It is not clear which of these turns may have driven the early articulatory preparation effect [35], but note that a stronger effect was found for short responses. For a better comparison to the present study it would be interesting to investigate question-answer pairs within spontaneous corpora only.

In contrast, the present results appear to be somewhat at odds with those of the study looking at breathing before answers to questions in spontaneous conversations between friends [33]. There, inbreaths before answers started most often around question offset. Although the inbreaths were not time locked to the response (only to question offset), such a timing appears later than in the present study (and [35]) and suggests that the inbreath was more-or-less tied to the response (assuming that the response in most cases came right after the inbreath). However, a first thing to keep in mind is that inbreaths occurred most often before long answers [33], whereas the early lip aperture differences [35] were mostly found before short answers. Second, on the one hand inbreaths may be regarded as a type of articulatory preparation, but on the other hand they may still behave differently to other types (such as lip openings and tongue movements). Given that inbreaths are often audible, taking an inbreath in preparation for speech, could be heard by one’s interlocutor and could thus be taken as a signal indicating an upcoming turn, or one’s intention to take a turn. Thus, because of the more ‘on record’ nature of inbreaths, it may be that upcoming speakers are more reluctant to take an inbreath long before their response, because it may seem to ‘interrupt’ the current turn, or signal impatience. Incidentally, mouth openings may also be *seen* by other interlocutors as indications that one wants to speak, whereas covert preparatory tongue movements may be less visible (and audible) by others.

More generally, as alluded to a few times now, the exact timing of the processes involved in response planning in all likelihood depends on several contextual factors. First, the amount of available planning time during the current turn is of course crucial. If there is a lot of time (i.e., the gist of the turn is clear very early on and the turn is of some length), planning presumably starts early and may be finished sooner. At the same time, this means more of the planning is done in overlap, which means this part of the planning is probably delayed, as shown by the present and earlier results (e.g., [17, 23]). How much the production planning process is slowed down by such overlap may depend on several factors as well. For example, one study suggests that the relative effort put into language comprehension versus language production processes may depend on individual differences [18], which may relate to personality characteristics such as extraversion. Within individuals, this balance may also change based on characteristics of the conversation or the type of relationship between communication partners (e.g., familiarity, friendship status, or dominance versus equality). Another important factor may be the type of turn that is planned (e.g., its length, complexity, and the amount of priming from context), and the extent to which, especially longer turns are being planned incrementally (see, e.g., [48]). Within some earlier studies, the variability in planned turns was very high (e.g., [35], and to a lesser extent [33]). Even in the present study, although the stimuli were highly controlled and similar in structure, there may still be variability between items (e.g., in how easily the answer was retrievable) and between participants in when the stage of articulatory preparation was reached (and whether it was reached before question offset). Although the differences between the two conditions in tongue movement timings, time-locked to response onset, were clearly reliable, the effect visible in Fig 6 at the base of the rise appears quite small. Such a relatively subtle effect suggests that the items varied a lot in when exactly articulatory preparation took place, such that it overlapped with the incoming turn only in a subset of early-planning items, or the effect was exhibited by only a subset of participants (or both). It is also possible that in other early-planning items, the stages up to articulatory preparation were simply not finished in time for articulatory preparation to start earlier than in the late-planning condition.

For future research, corpus studies investigating natural conversation (such as [33, 35]) are certainly relevant, but it is also important to investigate more systematically and experimentally how much production planning is slowed down by overlapping speech (relative to planning ‘in the clear’) in different circumstances. This may be done on the one hand by relatively ‘low-level’, carefully controlled experiments investigating the consequences of different parameters on the timing of (different phases of) speech planning. On the other hand, researchers could also employ more interactive paradigms, such as in the present study, varying the circumstances of the interaction. Specifically, it would be interesting to directly compare the timing of different aspects of articulatory preparation depending on how accessible they are to interlocutors–breathing and lip movements perhaps being more subject to suppression and delay due to their ‘on record’ character.

We argue that the (perceived) interactive aspect is especially relevant to include in such experiments because it may quantitatively or qualitatively change the processes involved. In other aspects, recreating natural conversation in controlled circumstances is also challenging. For example, the present study did not include visual aspects of interaction (e.g., gestures and facial expressions) and was restricted to question-answer sequences. Although phone conversations, and questions followed by socially expected answers are an important part of natural conversation, future research should broaden its scope to face-to-face interaction and other types of conversational sequences [49] as well.

## Conclusion

When taking turns in conversation, communicators have to be listeners and speakers at the same time, since they often start planning their own response while still listening to the previous turn. However, this dual tasking is not without cost. The present study showed that early planning can proceed all the way up to articulatory preparation in the form of tongue movements before speech onset, and arrive there some time (in the present study up to two seconds) before the response starts. This is in accordance with earlier observational work suggesting that articulatory preparation is not strictly tied to the response itself. However, we also found no evidence for early articulatory preparation, which is in accordance with the body of evidence showing that the planning process appears drawn out and less efficient when performed in overlap with understanding of the ongoing turn.

## Supporting information

S1 Text Table A with list of stimuli and Section B with results of the control analyses. (PDF)
