# Supplementary material for: Ultrasound measurements of interactive turn-taking in question-answer sequences: Articulatory preparation is delayed but not tied to the response
Source: PLoS One. 2023 Jul 5;18(7):e0276470. doi: 10.1371/journal.pone.0276470 (PMC10321606; doi:10.1371/journal.pone.0276470)
Supplement: S1 Text — (PDF) [file pone.0276470.s001.pdf]

Supporting Information

Ultrasound measurements of interactive turn-taking in question-answer sequences: Articulatory preparation is delayed but not tied to the response

Table A. List of Stimuli. Questions (in Dutch) used as experimental stimuli in the experiment (taken from Bögels et al., 2015), followed by the correct answer in brackets. Each question is first presented in the early-planning version, then in the late-planning version. Words in bold are the critical words that give the information that is needed to answer the questions in the early-planning questions, and the equivalent (control) words in the late-planning questions.

Nr	Question (correct answer)
1	15 is de **helft** van dit ronde getal. (30)
1	Van dit ronde **getal** is de helft 15. (30)
2	Als je rood en geel **mengt**, welke kleur krijg je dan? (oranje)
2	Welke kleur **krijg** je bij het mengen van rood en geel? (oranje)
3	De opvolger van de **videoband** is deze multimediadrager. (DVD)
3	Deze **multimediadrager** is de opvolger van de videoband. (DVD)
4	Deze grootste studentenvereniging van **Nijmegen** is tevens de oudste. (Carolus Magnus)
4	Van **Nijmegen** is dit de grootste en oudste studentenvereniging. (Carolus Magnus)
5	Een **ijsberg** speelt een belangrijke rol in deze met vele oscars bekroonde film. (Titanic)
5	Een belangrijke rol in deze met vele oscars bekroonde **film** is voor een ijsberg. (Titanic)
6	Elk GSM-nummer **begint** in Nederland hiermee. (06)
6	Hiermee begint in **Nederland** elk GSM-nummer. (06)

7 Haat is het **tegenovergestelde** van dit abstracte begrip. (liefde)

7 Het tegenovergestelde van dit abstracte **begrip** is haat. (liefde)

8 Het Engelse woord voor **liefde** is dit frequente woord. (love)

8 Dit frequente **woord** is het Engelse woord voor liefde. (love)

9 Hoe heet de partner van **Bassie** in de bekende serie? (Adriaan)

9 In de bekende **serie**, hoe heet de partner van Bassie? (Adriaan)

10 Hoe heet het vriendje van **Ieniemienie** in het televisieprogramma? (Tommie)

10 In het **televisieprogramma**, hoe heet het vriendje van Ieniemienie? (Tommie)

11 Hoeveel **nieren** heeft een gezond mens? (2)

11 Een gezond **mens** heeft zoveel nieren. (2)

12 Hoeveel **provincies** heeft Nederland sinds 1986? (12)

12 Sinds **1986** heeft Nederland zoveel provincies. (12)

13 Hoeveel **vingers** heeft een gezond mens? (10)

13 Een gezond **mens** heeft zoveel vingers. (10)

14 In plaats van het Franse **jus d'orange** gebruikt men ook dit Nederlandse woord. (sinaasappelsap)

14 Dit **Nederlandse** woord gebruikt men ook in plaats van het Franse jus d'orange. (sinaasappelsap)

15 Leven is het **tegenovergestelde** van dit abstracte begrip. (dood)

15 Het tegenovergestelde van dit abstracte **begrip** is leven. (dood)

16 Madrid is de **hoofdstad** van dit Europese land. (Spanje)

16 Van welk Europese **land** is de hoofdstad Madrid? (Spanje)

17 Op **vijf december** viert Nederland deze feestdag. (Sinterklaas)

17 Nederland viert deze **feestdag** op vijf december. (Sinterklaas)

18 Rome is de **hoofdstad** van dit Europese land. (Italië)

18 Van welk Europese **land** is de hoofdstad Rome? (Italië)

19 SP is de **afkorting** waarmee deze organisatie wordt aangeduid. (Socialistische Partij)

19 Deze organisatie wordt **aangeduid** met de afkorting SP. (socialistische partij)

20 Van welke popgroep was **Victoria Beckham** één van de bekende leden? (Spice Girls)

20 Van welke popgroep was 1 van de **bekende** leden Victoria Beckham? (Spice Girls)

21 Wandelen is een **synoniem** van dit bekende werkwoord. (lopen)

21 Een synoniem van dit bekende **werkwoord** is wandelen. (lopen)

22 Wat is de taal die in **Australië** door de inwoners wordt gesproken? (Engels)

22 Wat is de taal die wordt **gesproken** door de inwoners van Australië? (Engels)

23 Wat voor een dier is **Dombo** in de gelijknamige Disneyfilm? (olifant)

23 In de gelijknamige **Disneyfilm**, wat voor een dier is Dombo? (olifant)

24 Welk dier, dat het jong van een **schaap** is, vinden mensen schattig? (lam)

24 Welk dier, dat mensen **schattig** vinden, is het jong van een schaap? (lam)

25 Welk hoofddeksel, traditioneel gedragen door een **koning**, is alleen voor speciale gelegenheden? (kroon)

25 Welk hoofddeksel, alleen voor speciale **gelegenheden**, wordt traditioneel gedragen door een koning? (kroon)

26 Welk merk, vooral bekend om zijn **pindakaas**, komt uit Nederland? (Calvé)

26 Welk merk uit **Nederland** is vooral bekend om zijn pindakaas? (Calvé)

27 Welk product zit in alle **stamppotten** als belangrijk ingrediënt? (aardappel)

27 Welk product zit als belangrijk **ingrediënt** in elke stamppot? (aardappel)

28 Welk soort weer heeft **bliksem** als belangrijk onderdeel? (onweer)

28 Welk soort weer heeft als belangrijk **onderdeel** bliksem? (onweer)

29 Welk spel, waarin je **woorden** moet aanleggen, speel je op een gekleurd speelbord? (Scrabble)

29 In welk spel, op een **gekleurd** speelbord, moet je woorden aanleggen? (Scrabble)

30 Welke cabaretier, die het liedje **Flappie** zong, is erg bekend in Nederland? (Youp van 't Hek)

30 Welke cabaretier, die erg bekend is in **Nederland**, zong het liedje Flappie? (Youp van 't Hek)

31 Welke man, die de oprichter van **Apple** is, overleed enkele jaren geleden? (Steve Jobs)

31 Welke man, die enkele jaren **geleden** overleed, is de oprichter van Apple? (Steve Jobs)

32 Welke voornaam hoort bij **Van het Hek**, de beroemde cabaretier? (Youp)

32 Welke voornaam hoort bij de beroemde **cabaretier** Van het Hek? (Youp)

33 Welke woorden, die vaak aan het begin staan van een **sprookje**, zijn bekend bij iedereen? (er was eens)

33 Welke woorden, die bekend zijn bij **iedereen**, staan vaak aan het begin van een sprookje? (er was eens)

34 Wie krijgt een **chocoladefabriek** in het boek van een bekende Engelse schrijver? (Sjakie)

34 In het boek van een bekende Engelse **schrijver**, wie krijgt een chocoladefabriek? (Sjakie)

35 Zoveel **sterrenbeelden** onderscheiden we in Europa. (12)

35 In **Europa** onderscheiden we zoveel sterrenbeelden. (12)

36 Welk gebouw, gebruikt door **katholieken**, is voor iedereen toegankelijk? (kerk)

36 Welk gebouw, dat voor iedereen **toegankelijk** is, wordt gebruikt door katholieken? (kerk)

37 Welk land, bekend om zijn **pizza's**, ligt in Europa? (Italië)

37 Welk land, dat in Europa **ligt**, is bekend om zijn pizza's? (Italië)

38 Welk land, bekend om zijn **sushi**, ligt ver van Nederland? (Japan)

38 Welk land, dat ver van **Nederland** ligt, is bekend om zijn sushi? (Japan)

39 Welke schrijfster, bekend van de boeken van **Jip en Janneke**, heeft meer dan honderd boeken geschreven? (Annie M.G. Schmidt)

39 Welke schrijfster, die meer dan honderd **boeken** geschreven heeft, is bekend van de boeken van Jip en Janneke? (Annie M.G. Schmidt)

40 Welk schoolvak, met als onderdeel **topografie**, krijg je op de middelbare school? (aardrijkskunde)

40 Van welk schoolvak, dat je krijgt op de middelbare **school**, is een onderdeel topografie? (aardrijkskunde)

41 Welke stof zit in **sigaretten** als effectief element? (nicotine)

41 Welke stof zit als effectief **element** in sigaretten? (nicotine)

42 Welke stof zit in **koffie** als effectief element? (cafeïne)

42 Welke stof zit als effectief **element** in koffie? (cafeïne)

43 Welke zangeres had met **'Valerie'** in 2007 een grote hit? (Amy Winehouse)

43 Welke zangeres had in 2007 een grote **hit** met 'Valerie'? (Amy Winehouse)

44 In welke films speelt **Frodo** de belangrijkste rol? (Lord of the Rings)

44 In welke films is de belangrijkste **rol** voor Frodo? (Lord of the Rings)

45 Welk toetje, dat **bevroren** is, komt in verschillende smaken voor? (ijs)

45 Welk toetje, dat in verschillende **smaken** voorkomt, is bevroren? (ijs)

46 Welke naam, die soms nog wordt gebruikt als aanduiding voor **zwarte** mensen, wordt als denigrerend beschouwd? (neger)

46 Welke naam, die als **denigrerend** wordt beschouwd, wordt soms nog gebruikt als aanduiding voor zwarte mensen? (neger)

47 Voor welke groente is een belangrijke rol in de tekenfilm **Popeye**, waarin de gelijknamige zeeman de hoofdrol speelt? (spinazie)

47 In de tekenfilm Popeye, waarin de gelijknamige zeeman de hoofdrol **speelt**, is een belangrijke rol voor deze groente. (spinazie)

48 Welke afkorting, waarmee de belangrijkste Nederlandse **vliegmaatschappij** wordt aangeduid, bestaat uit drie letters? (KLM)

48 Wat is de afkorting, bestaande uit drie **letters**, voor de belangrijkste Nederlandse vliegmaatschappij? (KLM)

49 Welk werelddeel, waarin de meeste mensen **Spaans** spreken, heeft een oppervlakte van meer dan 17 miljoen vierkante kilometer? (Zuid-Amerika)

49 In welk werelddeel, dat een oppervlakte heeft van meer dan 17 miljoen vierkante **kilometer**, spreken de meeste mensen Spaans? (Zuid-Amerika)

50 Welke dieren, waarvan het geluid **balken** heet, hebben een onopvallende kleur? (ezels)

50 Van welke dieren, met een onopvallende **kleur**, heet het geluid balken? (ezels)

51 Welk karakter, ook wel **007**, komt voor in de bekende films? (James Bond)

51 Welk karakter uit de bekende **films** heet ook wel 007? (James Bond)

52 Welke dieren, die **post** kunnen bezorgen, zijn een belangrijk symbool in de kunst? (duiven)

52 Welke dieren, die een belangrijk symbool in de **kunst** zijn, kunnen ook post bezorgen? (duiven)

53 De zeven dwergen spelen in dit **sprookje** een belangrijke rol. (Sneeuwwitje)

53 Een belangrijke rol in dit **sprookje** is voor de zeven dwergen. (Sneeuwwitje)

54 Welke maand, die op mei **volgt**, heeft een naam die uit het Latijn komt? (juni)

54 Welke maand, die een naam heeft die uit het **Latijn** komt, volgt op mei? (juni)

55 Welke maand, waarin veel **feestdagen** zijn, heeft een Latijnse naam? (december)

55 In welke maand, met een Latijnse **naam**, zijn er veel feestdagen? (december)

56 Welke dieren, die **miauwen**, kunnen goed in het donker zien? (katten/poezen)

56 Welke dieren, die goed in het donker kunnen **zien**, miauwen? (katten/poezen)

57 Welk voorwerp dat meestal gebruikt wordt om te **knippen**, vind je in een klaslokaal? (schaar)

57 Welk voorwerp, dat je vindt in een **klaslokaal**, wordt meestal gebruikt om te knippen? (schaar)

58 Welke rivier, die door **Egypte** stroomt, is zowel lang als breed? (Nijl)

58 Welke rivier, die zowel lang als **breed** is, stroomt door Egypte? (Nijl)

9

Section B. Results control analyses

Below, we present the statistical results for all three ultrasound analyses (with different time-locking points) reported in the manuscript, for three different subsets of the data: (1) 18 participants with good-contrast ultrasound images (see *Data analysis* within *Methods*); (2) 19 participants with good-coverage ultrasound images (see *Data analysis* within *Methods*); (3) 17 participants who believed that the questions were asked live by the experimenter (see *Questionnaire* within *Results*).

Question offset. For all three control analyses, the results for time-locking to question offset were qualitatively very similar to the results for the whole sample. The analysis with 18 participants with good-contrast ultrasound images showed a positive cluster between 884 ms before and 720 ms after question offset ($sumT = 875.4$, $p < .001$) and a negative cluster between 1048 and 2313 ms ($sumT = -365.6$, $p = .004$). The analysis with 19 participants with good-coverage ultrasound images showed a positive cluster between 873 ms before and 753 ms after question onset ($sumT = 1072.2$, $p < .001$) and a negative cluster between 1004 and 1931 ms ($sumT = -358.3$, $p = .004$). The analysis with 17 participants who believed the manipulation showed a positive cluster between 862 ms before and 840 ms after question onset ($sumT = 1075.0$, $p < .001$) and a negative cluster between 1069 and 1920 ms ($sumT = -293.8$, $p = .019$).

Early planning point. For all three control analyses, the results for the early planning point (time-locking to the onset of the critical word in the early-planning condition and an equivalent position in the middle of the question in the late-planning condition; i.e., TL2 in Fig 1) were qualitatively very similar to the results for the whole sample. The analysis with 18 participants with good-contrast ultrasound images showed a positive cluster between 2761 and 4583 ms ($sumT = 656.1$, $p < .001$). The analysis with 19 participants with good-coverage ultrasound images showed a positive cluster between 2204 and 4692 ms ($sumT = 994.6$, $p < .001$). The analysis with 17 participants who believed the manipulation showed a positive cluster between 2106 and 4681 ms ($sumT = 1012.8$, $p < .001$).

Speech onset. For all three control analyses, the results for the speech onset time-locking position were qualitatively similar to the results for the whole sample, although the effect became significant only later, probably due to a loss of power. The analysis with 18 participants with good-contrast ultrasound images showed a positive cluster between 1124 and 218 ms before speech onset (*sumT* = 334.3.1, p = .004). The analysis with 19 participants with good-coverage ultrasound images showed a positive cluster between 1855 and 142 ms before speech onset (*sumT* = 646.6, p < .001). The analysis with 17 participants who believed the manipulation showed a positive cluster between 1440 and 142 ms before speech onset (*sumT* = 611.3, p = .002).
